# Non-echo Planar Diffusion-Weighted Imaging in the Detection of Recurrent or Residual Cholesteatoma: A Systematic Review and Meta-Analysis of Diagnostic Studies

**DOI:** 10.7759/cureus.32127

**Published:** 2022-12-02

**Authors:** Hosam Amoodi, Abdelelah Mofti, Nawaf H Fatani, Hatem Alhatem, Ahmed Zabidi, Mohammad Ibrahim

**Affiliations:** 1 Otolaryngology-Head and Neck Surgery, University of Jeddah, Jeddah, SAU; 2 Otolaryngology-Head and Neck Surgery, Dr. Soliman Fakeeh Hospital, Jeddah, SAU; 3 Otorhinolaryngology-Head and Neck Surgery, King Abdullah Medical City, Jeddah, SAU; 4 Otolaryngology-Head and Neck Surgery, Imam Abdulrahman Bin Faisal Hospital, Riyadh, SAU

**Keywords:** non-epi, meta-analysis, diagnostic accuracy, second-look surgery, recurrent, cholesteatoma

## Abstract

We performed a systematic review and meta-analysis of patients with suspected recurrent cholesteatoma who underwent non-echo planar imaging (non-EPI) using diffusion-weighted magnetic resonance imaging (MRI), with surgery as the reference standard. We searched Medline, Google Scholar, and the Cochrane database for diagnostic test accuracy studies. The following prespecified subgroup analyses were performed: patient age, number of radiologists interpreting MRI, study design, and risk of bias. We used a bivariate model using a generalized linear mixed model to pool accuracies. Of the 460 records identified, 32 studies were included, of which 50% (16/32) were low risk of bias. The overall pooled sensitivity was 92.2% (95% CI 87.3-95.3%), and specificity was 91.7% (85.2-95.5%). The positive likelihood ratio was 11.1 (4.5-17.8), and the negative likelihood ratio was 0.09 (0.04-0.13). The pooled diagnostic odds ratio was 130.3 (20.5-240). Heterogeneity was moderate on visual inspection of the hierarchical summary receiver operating characteristic curve. Subgroup analyses showed prospective studies reporting higher accuracies (p=0.027), which were driven by higher specificity (prospective 93.1% (88.4-96.0%) versus retrospective 81.2% (81.0-81.4%)). There was no difference in subgroups comparing patient age (p=0.693), number of radiologists interpreting MRI (p=0.503), or risk of bias (p=0.074). No publication bias was detected (p=0.98). In conclusion, non-EPI is a highly sensitive and specific diagnostic test able to identify recurrent cholesteatomas of moderate to large sizes. This test can be considered a non-invasive alternative to second-look surgery.

## Introduction and background

A cholesteatoma is a congenital or acquired middle ear lesion composed of the stratified keratinizing epithelium [1]. Despite primary surgical resection being generally successful in alleviating the initial patient presentation, remnants of keratinized epithelium can lead to recurrent cholesteatoma [2]. This becomes challenging for otolaryngologists at follow-up examinations as the surgical approach may disfigure anatomy and have recurrent cholesteatoma in areas not visible on otoscopy [3]. Therefore, second-look surgery is often performed to check for residual disease and is considered the golden standard for diagnosis [3]. However, with the exploratory nature of the second procedure, patients are once again subjected to the inherent risks of surgery.

Recently, diffusion-weighted imaging (DWI) using echo-planar and non-echo-planar sequences has emerged as an accurate and non-invasive diagnostic test for cholesteatoma. Non-echo planar imaging (non-EPI) has particularly demonstrated superiority in detecting cholesteatoma when compared to other sequences. By offering a non-invasive method of accurately identifying post-operative cholesteatoma, non-EPI offers a potential alternative to second-look surgery.

Currently, studies to date have investigated the diagnostic accuracy of non-EPI DWI; however, these studies have small sample sizes with variable results [4,5]. Whereas previous systematic reviews evaluated non-EPI [6], a considerable number of studies have been published, which warrants an up-to-date meta-analysis. Therefore, we performed a systematic review and meta-analysis evaluating the diagnostic accuracy of non-EPI for recurrent or residual cholesteatoma.

## Review

Materials and methods

The preferred reporting items for systematic reviews and meta-analyses (PRISMA) guidelines for diagnostic test accuracy (DTA) and the Cochrane Handbook for Systematic Reviews of DTA studies were followed for this systematic review [7]. We searched Medline, Google Scholar, and the Cochrane Library for patients of any age presenting with suspected recurrent or residual cholesteatoma who underwent non-EPI diffusion-weighted magnetic resonance imaging (MRI). Eligible studies required patients to undergo surgery as the reference standard. We excluded studies that combined non-EPI and post-contrast magnetic resonance sequences as the reference standard or that used other reference standards, such as otoscopy. If studies included both primary and recurrent cholesteatoma or various MRI sequences, they were included only if there was sufficient data granularity to permit the extraction of patients relevant to our research question. Studies were excluded if a contingency table for non-EPI DWI could not be constructed. In addition, we searched the references of all articles included as well as review articles that were identified in our initial search. We excluded non-English articles and studies that did not evaluate the diagnostic test accuracy of non-EPI DWI (e.g., interventional studies evaluating correlations between two tests). Institutional review board approval was not required as all the data was publically available.

Studies were screened independently and in duplicate by two investigators. Disagreements regarding inclusion were resolved through consensus. Risk of bias was performed using the quality assessment of diagnostic accuracy studies-2 (QUADAS-2) tool, which evaluated the risk of bias in four domains (patient selection, index test, reference standard, and flow and timing) and applicability in three domains (patient selection, index test, and reference standard) [8]. Data were extracted by two investigators independently and in duplicate. For each study, a 2×2 contingency table was constructed using the sensitivities or specificities provided in each study. We also extracted additional demographic information, such as the study title, year of publication, age of patients (adult or pediatric), study design (prospective or retrospective), number of radiologists interpreting the non-EPI, and the smallest size of cholesteatoma correctly diagnosed using the non-EPI.

Statistical analysis

We performed a meta-analysis of the diagnostic accuracies of each study using the bivariate model, which was fitted using a generalized linear mixed model (GLMM). Study-level data were fitted using random effects using the Laplace approximation. The likelihood ratios and diagnostic odds ratios were calculated from pooled estimates of sensitivity and specificity.

Heterogeneity was explored by first constructing a hierarchical summary receiver operating characteristic (HSROC) curve, which included both 95% confidence intervals and 95% prediction intervals. The magnitude of heterogeneity was evaluated by visually comparing the distance of these two intervals.

Given the suspected heterogeneity of studies, we performed the following prespecified subgroup analyses: patient age (adult versus pediatric), number of radiologists interpreting non-EPI DWI (single versus multiple), study design (prospective versus retrospective), and risk of bias (low risk versus high risk). These analyses were performed by constructing a GLMM with and without each covariate added, and then compared using a likelihood ratio test. A significant difference between models was explored by comparing the sensitivities and specificities separately to determine which accuracy variable contributed to an overall statistical difference between models.

Publication bias was evaluated using the Deeks model [9]. A funnel plot is constructed using the effective sample size of studies. A formal evaluation of publication bias was performed using the regression test.

Meta-analysis was performed using the LME4 package in R statistical software (version 4.1.2) (R Foundation for Statistical Computing, Vienna, Austria) [10]. The HSROC curve was constructed using the METANDI package in STATA (version 17.0) (StataCorp, Texas, USA) [11]. Publication bias was assessed using the MIDAS package in STATA [12]. Forest plots were created using Review Manager (version 5.4.1) (RevMan, Copenhagen, Denmark). A p-value less than 0.05 was considered statistically significant.

Results

**Figure 1:**
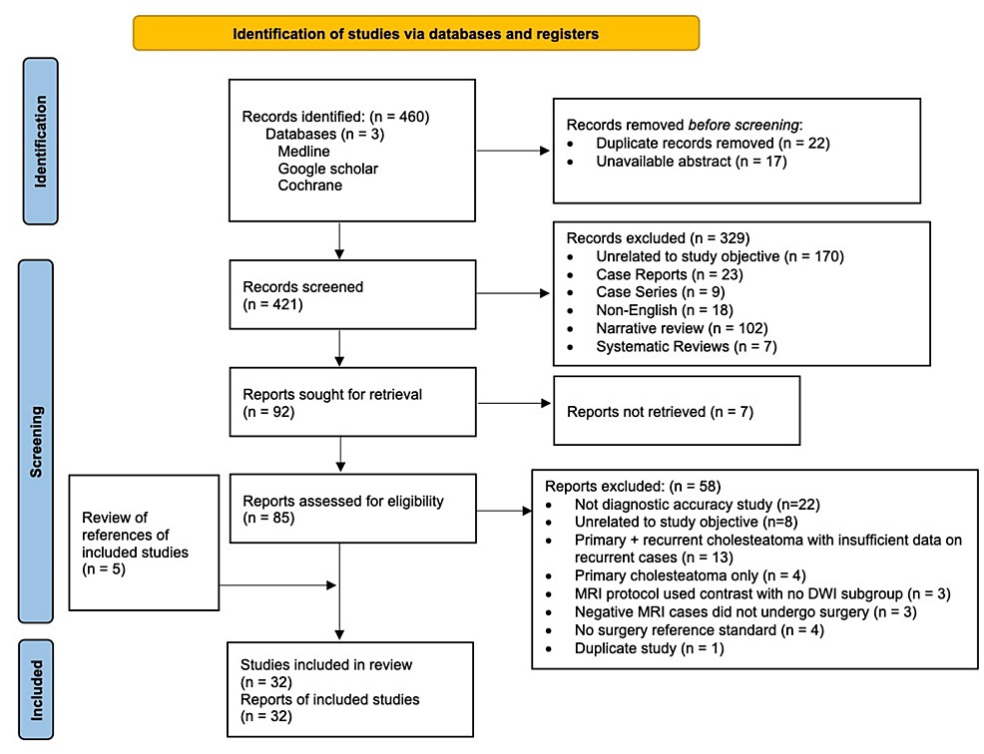
PRISMA flow chart showing the study selection process. PRISMA: preferred reporting items for systematic reviews and meta-analyses.

**Figure 2:**
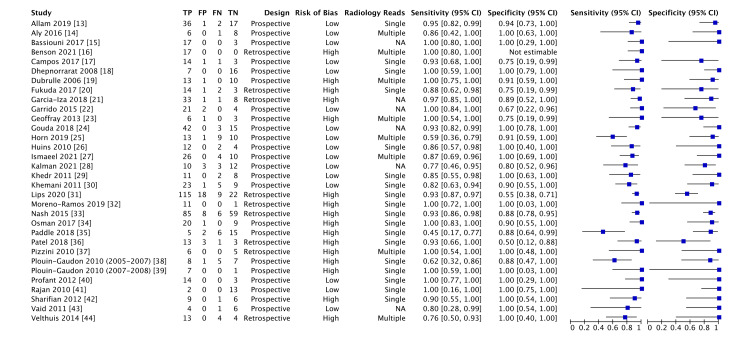
Characteristics, accuracies, and forest plots of the 32 included studies. Source: [13-44]. TP: true positive, FP: false positive, FN: false negative, TN: true negative. Note: This image is the author's own creation.

Our search initially identified 460 records, of which 421 unique records were screened and 32 were included [13-44]. Figure 1 shows details regarding study selection. Sixteen of 32 articles (50%) were rated as low risk of bias [13-15,17,18,22,24-30,40,41,43]. There were 23 prospective studies and nine retrospective studies. Of the studies reporting patient age, six included adults, one included both adults and children, and four included children. Of the studies reporting the number of radiologists interpreting the index test, eight used multiple radiologists interpreting each scan and 18 used a single radiologist. The median smallest cholesteatoma correctly identified using non-EPI was 3.0 mm, which ranged from 2.0 to 5.0 mm. Characteristics of included studies and their diagnostic accuracies are shown in Figure 2.

For the overall meta-analysis, including 32 studies, the pooled sensitivity was 92.2% (95% CI 87.3-95.3%), and the pooled specificity was 91.7% (95% CI 85.2-95.5%). The corresponding positive likelihood ratio was 11.1 (95% CI 4.5-17.8), and the negative likelihood ratio was 0.09 (95% CI 0.04-0.13). The diagnostic odds ratio was 130.3 (95% CI 20.5-240). Table 1 summarizes the sensitivities, specificities, positive likelihood ratios, and negative likelihood ratios of all meta-analyses.

**Table 1:** Pooled diagnostic accuracies of non-EPI DWI. EPI: echo-planar imaging; DWI: diffusion-weighted imaging; LR: likelihood ratio; MRI: magnetic resonance imaging.

Meta-analysis	Sensitivity (%)	Specificity (%)	LR+	LR–	P-value
Overall	92.2 (87.3-95.3)	91.7 (85.2-95.5)	11.1 (4.5-17.8)	0.09 (0.04-0.13)	Not applicable
Age					0.693
Adult	94.4 (82.3-98.4)	93.0 (78.7-98.0)	13.5 (0-29.9)	0.06 (0-0.13)	
Pediatric	98.5 (0.0-99.9)	93.0 (0.71-98.6)	14.2 (0-36.6)	0.02 (0-0.16)	
Radiologists interpreting MRI					0.503
Single	91.4 (84.8-95.3)	90.6 (80.7-95.7)	9.7 (2.3-17.2)	0.09 (0.04-0.15)	
Multiple	92.1 (72.0-98.2)	94.2 (83.4-98.1)	16.0 (0-33.6)	0.08 (0-0.20)	
Study design					0.027
Prospective	91.5 (84.3-95.5)	93.1 (88.4-96.0)	13.2 (6.1-20.4)	0.09 (0.03-0.15)	
Retrospective	93.1 (93.0-93.1)	81.2 (81.0-81.4)	5.0 (4.9-5.0)	0.09 (0.08-0.09)	
Risk of bias					0.074
High	93.9 (85.5-97.6)	85.2 (74.1-92.1)	6.3 (2.5-10.1)	0.07 (0.01-0.14)	
Low	90.6 (83.8-94.7)	95.5 (86.7-98.5)	20.0 (0-42.5)	0.10 (0.04-0.15)	

**Figure 3:**
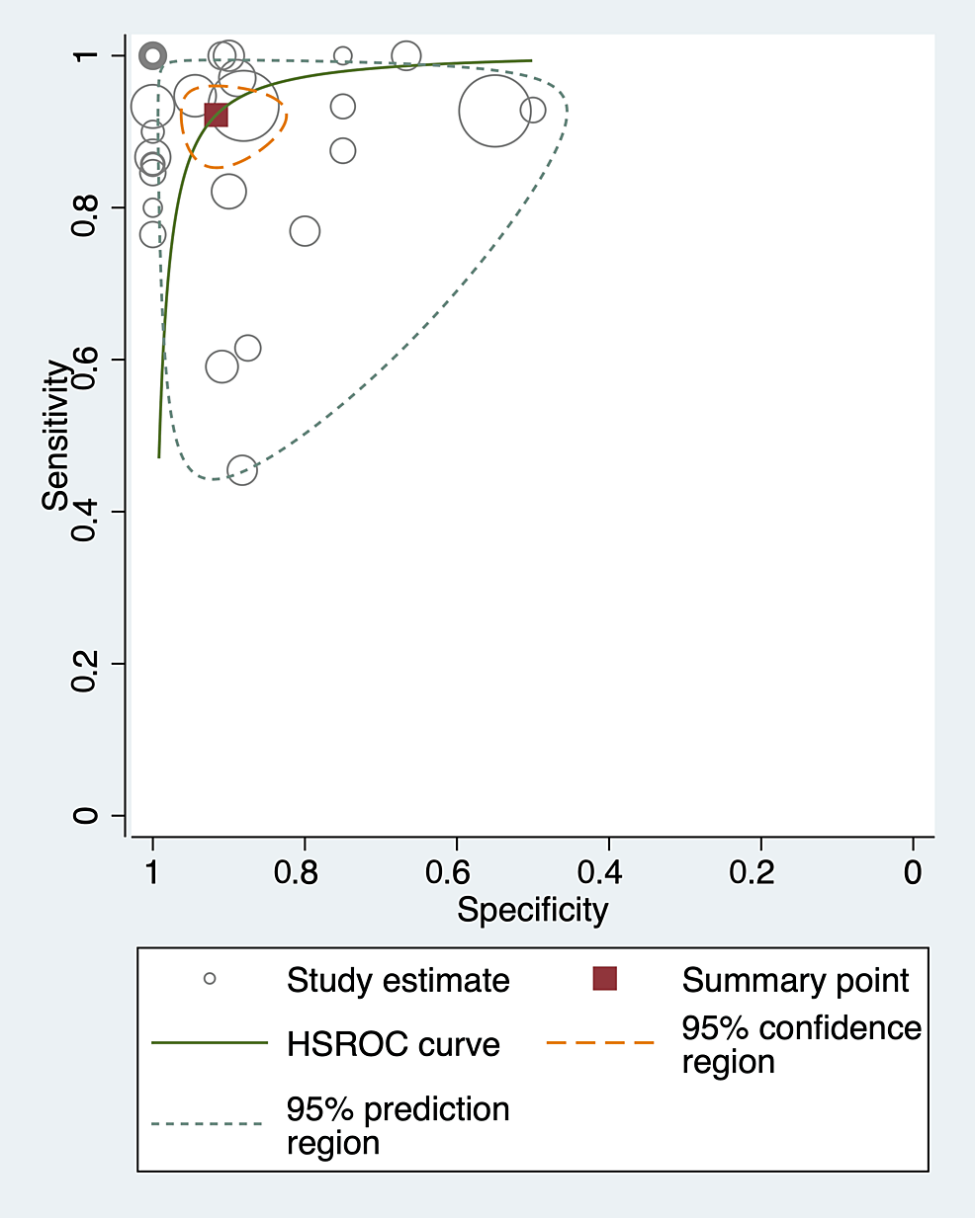
Hierarchical summary receiver operating characteristic curve showing a pooled sensitivity of 92.2% and a pooled specificity of 91.7%. The 95% confidence intervals and 95% prediction intervals show a moderate distance between one another, indicating moderate heterogeneity. HSROC: hierarchical summary receiver operating characteristic curve. Note: This image is the author's own creation.

Heterogeneity was explored using the HSROC curve (Figure 3), which shows the pooled summary point and individual studies depicted as bubbles. Individual studies generally showed proximity to the summary point. On visual inspection, the 95% confidence interval was a short distance from the summary point. There was a moderate distance between the 95% confidence intervals and the 95% prediction intervals, suggesting a moderate level of heterogeneity.

Heterogeneity was explored through subgroup analyses. We found a significant difference in diagnostic accuracy between prospective and retrospective study designs (p=0.027). Specifically, prospective studies showed a sensitivity of 91.5% (95% CI: 84.3-95.5%) and a specificity of 93.1% (95% CI: 88.4-96.0%). Retrospective studies showed a sensitivity of 93.1% (95% CI: 93.0-93.1%) and a specificity of 81.2% (95% CI: 81.0-81.4%). Subgroup analysis comparing only sensitivities between these study designs showed no significant difference (p=0.292); however, subgroup analysis comparing specificities showed a significant difference (p=0.015), suggesting the difference in models was driven by differences in specificities.

There was no significant difference in diagnostic accuracy between the low-risk of bias studies and the high-risk of bias studies (p=0.074). Specifically, studies rated as low risk of bias showed a sensitivity of 90.6% (95% CI: 83.8-94.7%) and specificity of 95.5% (95% CI: 86.7-98.5%). Studies rated high-risk of bias showed a sensitivity of 93.9% (95% CI: 85.5-97.6%) and specificity of 85.2% (95% CI: 74.1-92.1%). There was no significant difference in diagnostic accuracy when comparing single versus multiple radiologists interpreting the non-EPI DWI (p=0.503). Specifically, single radiologists showed a sensitivity of 91.4% (95% CI: 84.8-95.3%) and a specificity of 90.6% (95% CI: 80.7-95.7%). Multiple radiologists interpreting non-EPI DWI showed a sensitivity of 92.1 (95% CI: 72.0-98.2%) and specificity of 94.2% (95% CI: 83.4-98.1%). Finally, there was no significant difference between adult patients and pediatric patients (p=0.693). Non-EPI showed a sensitivity of 94.4% (82.3-98.4%) and a specificity of 93.0% (78.7-98.0%) in adults. In pediatric patients, the sensitivity was 98.5% (0.0-99.9%), and the specificity was 93.0% (0.71-98.6%). 

**Figure 4:**
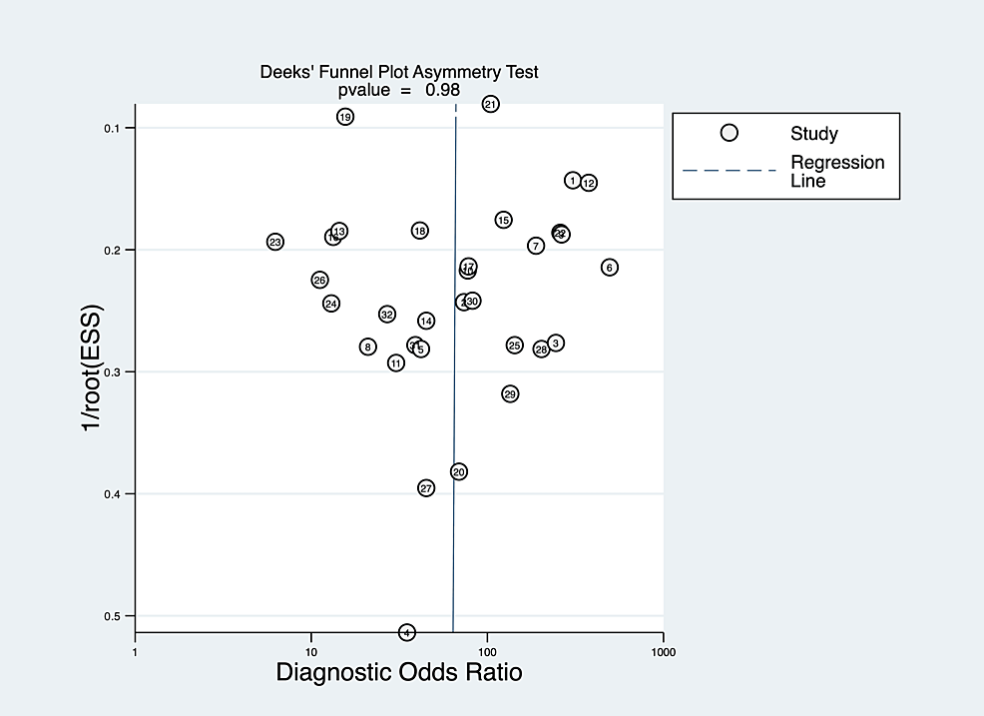
Funnel plot constructed using Deeks model, showing no asymmetry on visual inspection or regression test (p=0.98). ESS: effective sample size. Note: This image is the author's own creation.

There was no evidence of publication bias on visual inspection of the funnel plot (Figure 4). Formal analysis using Deeks’ regression test did not detect publication bias (p=0.98). 

Discussion

This systematic review and meta-analysis demonstrated that non-EPI DWI is a sensitive and specific diagnostic test for recurrent cholesteatoma. Our pooled results identified a low negative likelihood ratio of 0.09, which can accurately rule out recurrent cholesteatoma and spare patients the risks associated with surgery. A high positive likelihood ratio of 11.1 permits the selection of patients with recurrent cholesteatoma requiring additional surgery with the plan for resection. In addition, in these patients, surgeons have access to pre-operative information obtained from MRI for surgical planning. The results of our meta-analysis were consistent between evaluated subgroups, except for prospective studies, which showed higher accuracy than retrospective studies.

A positive result on non-EPI DWI warrants a second look or revision surgery in patients with suspected recurrent cholesteatoma. Accordingly, false-positive cases may confer unnecessary exposure to surgery for a patient. A review article evaluating false positive results for cholesteatoma suggested several potential etiologies: bone graft sealant used in primary surgery, cholesterol granuloma, and granulation tissue with fibrosis [45]. Locketz and colleagues reported a false positive using periodically rotated overlapping parallel lines with enhanced reconstruction (PROPELLER) DW MRI, which after the surgical examination was found to be secondary to the tragal cartilage used in the primary surgery [46]. Horn and colleagues similarly reported a false-positive case that was later determined to be cartilage material [25]. Plouin-Gaudon and colleagues also reported a false-positive case suspected to be due to motion artifacts and metallic dental braces [38]. Awareness of these abnormal materials in locations where a cholesteatoma is suspected may improve non-EPI diagnostic accuracy by communicating them to the interpreting radiologist.

A negative result on a non-EPI DWI can spare a patient the risks associated with surgery. Accordingly, false-negative cases may erroneously categorize patients with cholesteatoma as those not requiring surgery. In our systematic review, we found many studies reporting the smallest size of cholesteatoma correctly diagnosed on non-EPI DWI. Similar to the literature commonly reporting 3 mm as a cutoff, we found that when evaluating all included studies, the median smallest size of cholesteatoma correctly diagnosed was also 3 mm. This cutoff was reported in nine of our included studies [15,18,26,29,30,32,39,40,41]. The smallest cholesteatoma correctly identified was 2.0 mm, which among other studies, was reported by Horn and colleagues and measured intraoperatively [25]. Given this trend of smaller cholesteatomas being more likely to be missed radiologically, a common reason for false negatives was small cholesteatoma size. For instance, Nash and colleagues reported six false negatives in their study, which they mainly attributed to small points of disease [33]. From a clinical perspective, it is uncertain whether initially missing a small cholesteatoma can result in significant harm to patients. Accordingly, clinicians may consider repeat non-EPI in patients suspected to have recurrent cholesteatoma with initial imaging showing a negative result.

The learning curve associated with interpreting non-EPI may also explain some heterogeneity in our meta-analysis. This also may contribute to false negatives and false positives identified in some studies. While we were unable to formally evaluate this factor in our analysis, we did examine the different diagnostic accuracies when there were single versus multiple radiologists interpreting the scan. Despite not being statistically significant, in patients with suspected cholesteatoma, those who had multiple radiologists interpreting the scan had a higher magnitude of sensitivity (92.1% versus 91.4%) and specificity (94.2% versus 94.2%). This suggests that a collaborative effort among radiologists may assist in correctly evaluating some scans that may otherwise have been declared falsely positive given specific imaging artifacts. Given that the radiologists evaluating the MRIs in our included studies were fellowship-trained, the diagnostic accuracy of non-EPI DWI may have lower generalizability to radiologists without such training or experience.

Although our study supports the high diagnostic accuracy of non-EPI, we were unable to perform a formal cost comparison. Generally, the cost of surgical intervention is more expensive, time-consuming, resource intensive, and lengthy for the patient or healthcare system. Accordingly, the results of a cost comparison must be considered. Referencing the work conducted by Choi and colleagues in 2019 in Canada showed through a probabilistic sensitivity analysis that the mean cost difference between second-look surgery and non-EPI DWI was CAD 390.66 less for non-EPI DWI [47]. Therefore, the results of our study, in conjunction with results extrapolated from other studies, suggest that this diagnostic test may be beneficial from a resource perspective. However, these results are not generalized to patients with contraindications to MRI imaging modalities.

Our study has several strengths. First, our analysis was performed according to recommendations in the Cochrane Handbook for Systematic Reviews of diagnostic test accuracy studies. Our bivariate analysis accounted for the relationship between sensitivity and specificity within each study and permitted robust calculations of positive and negative likelihood ratios. Second, we evaluated several important clinical and methodological factors through a prespecified subgroup analysis, which explained some of the heterogeneity we encountered. Third, we only included patients who were suspected of recurrent or residual cholesteatoma, which allows for generating conclusions about whether second-look surgery is warranted.

Nevertheless, this systematic review has several limitations. First, we were unable to stratify patients into those who had recurrent versus those who had residual disease. This was not possible given the insufficient granularity of the data reported in individual studies. Second, some of the studies included were small and are prone to influence the overall pooled estimates should only one or two patients have a false result. This limitation was minimized as the meta-analysis was weighted, which is related to the sample size. Finally, some of the included studies were retrospective, which may result in selection bias and knowledge of the index test results when evaluating the reference standard. We addressed this limitation by performing a risk of bias analysis using the QUADAS-2 tool, which considered this limitation when evaluating each study. 

## Conclusions

This systematic review provides a comprehensive overview of the diagnostic accuracy of non-EPI DWI for recurrent or residual cholesteatoma, using surgery as the reference standard. Non-EPI is a highly sensitive and specific imaging test, able to rule in and rule out recurrent or residual cholesteatoma. The smallest cholesteatoma correctly identified by non-EPI is commonly reported to be 3 mm. Accordingly, clinicians may consider this imaging modality as an alternative to second-look surgery to rule out cholesteatomas of moderate to large sizes. Future studies evaluating the clinical impact of false negatives on non-EPI are warranted.
